# Advice to Young Doctors—Should the Young Doctor Marry?

**Published:** 1905-02

**Authors:** 


ADVICE TO YOUNG DOCTORS.—SHOULD THE YOUNG DOCTOR MARRY?

When the young doctor is settled in practice—perhaps before—the question of marriage will in all probability arise, and in settling it some matters should be considered.

Science is a jealous mistress and demands of its votaries unremitting attention, and no department of scientific work is more exacting or gives larger rewards for labor than medicine. The demands for time and study are great, and no young man can afford to neglect these obligations, if he wishes to attain success in his chosen profession. Nothing must be allowed to come between him and the goal for which he strives. All the powers of his intellect are needed, and this is more especially so in the early days of his career. It is at this time that the foundations for all future work and usefulness are laid. If a wife is to come between the young doctor and his work it were better that he should defer marriage until he is well established at least.

Firmly believing the expressions of the preceding paragraph, we unhesitatingly express the opinion that the young doctor should marry. Man is a social being; he demands, yea needs, the ties of social life; he craves sympathy in his labors, or rather a consciousness of a sympathetic feeling on the part of some one for his work; there is encouragement in the chance to discuss the matters that burden his mind and soul with a kindred being; there must be some one to whom he feels free to unburden himself; a confidant in whom he can place the most implicit trust; a companion of whom he does not tire; these should all be found in a wife; and no one needs them more than the doctor.

To advise one in the kind of a woman to select for a wife would be presumptious. Love should be the ruling element in marriage, and Cupid has a habit of disregarding all considerations when he draws his bow. A homily on love and marriage is hardly a suitable theme for a medical journal; still it may not be amiss to say that there is no subject more important than the preservation of the purity of our homes, the foundation of all civil and religious liberty, and happy homes are founded only upon love. The doctor as the State's best citizen, as a law-abiding man, as a leader in social life and advanced thinking, should give his example and influence to the preservation of the home.

The doctor's wife may be a hindrance to him in his efforts to build or hold a practice, a veritable millstone about his neck, or she may be a helpmeet to him worthy to share the emoluments of his labors or the honors of his fame. To describe the ideal doctor's wife would be as hard a task as to describe the ideal doctor, and only some of the ways in which she can aid her husband in his studies and practice will be indicated.

The first consideration is that she shall have a sympathetic interest in his work, in his studies, in his patients, in his success and in his failures. This is a prerequisite. Without it, it were better that she should know as little as possible of his work. With it, the doctor can confidently confer with her, tell her of his trials, share with her the pleasures of his success, and be sure of a faithful coadjutator in his plans.

There are many skeletons in the closet that will come to the doctor's knowledge. Some he will realize that he can not in justice to himself, his patient and his profession divulge even to his wife. If she is discreet and can be trusted with a secret (and women *can* be trusted with a secret) there are others he may discuss with advantage with her. Rest assured, though, that the doctor with a tattling wife will lose many calls he would otherwise receive. This is more pronounced in obstetrical and gynecological work, perhaps, than in any other. The doctor is called and ascertains that Mrs. So and So is in the first months of pregnancy. He tells his wife, she tells her sister or mother or best friend, or perhaps all of them, and in a few days it is generally known, to the chagrin and perhaps anger of the patient. The wife must be as discreet in speaking of her husband's patients as he is himself. If she can not be, the doctor must not confide in her.

The doctor's social circle or the social sphere in which the wife moves may be entirely separate and distinct from that of his practice. There will always be part of his clientele of a lower order of social life than hers, and if she is to be a helpmeet to him she will often be thrown in contact with members of this class. Much tact may be, and very often is, required to prevent a false impression on the minds of the visitors. Practically, they are looking for some slight, or something to criticize, and to put them at ease and create a good impression without overdoing the matter is no easy task. Much of the young doctor's work and a good percentage of his income are derived from this class, and it is well to conciliate it. A repellant manner or haughty demeanor on the part of the wife may drive a prospective patient and those of his friends whom he can influence to another physician.

Visits to his patients when sick are to be made with the greatest circumspection. Too much visiting to the sick is tolerated under the best circumstances, and a doctor's wife visiting the sick, even though her husband's patients, smacks very much of an appearance to curry favor, to seek patronage. The wife must be as ethical as her husband. A visit with the husband, or to a close friend, or to aid in the nursing may be permitted, but with the qualification that she goes as a neighbor or friend and not as the doctor's wife. For a doctor's wife to visit the patient of another physician is very questionable, and should be done only under exceptionable circumstances. If done at all no effort should be made to ascertain the line of treatment pursued, and suggestions, remarks or criticisms in regard to the case must be scrupulously avoided.

The every-day life of the doctor in commonplace material things can be much benefited by a wife's aid. In our observations the doctor's office is none too well kept and a woman's touch here and there daily will not come amiss. With little instruction the care of the instruments may be intrusted to the wife. The poor bookkeeping and collecting of the doctor is proverbial, and if this is turned over to the wife with full powers given to send out bills monthly and write urgent letters to delinquents the exchequer would not suffer. Women make excellent microscopists, and can easily learn to make the more simple urinary and blood analyses. The keeping and filing of papers and journals, the writing up of histories in the case book, attending to the correspondence, are some of the ways a wife may render material aid to the young doctor until his practice attains such proportions as to justify a trained nurse or office assistant.

But it is in the home life of the doctor the wife's influence is most essential and it is here that it is either a blessing or a curse. When the day's work is done and the nervous tension is relaxed, the tired brain and exhausted body seek rest, and the perplexities of life are cast aside, the office of the wife is to minister not only to the bodily necessities, but to the mental state as well. It is hers to cheer the drooping spirits, to encourage, to soothe—sympathize if need be, to rejoice if possible. This, it may be claimed, is but the office and duty of the wife of any man, whatever his calling. If so, no one needs it, or will appreciate it more, than the doctor.

And if she gives this aid and comfort, and in later years becomes interested in aid societies, goes out among highways and hedges, or back street and dirty alleys, to seek the maimed and halt and blind, and having found them, sends forthwith for you to minister to their needs, it will add to your peace of mind and material welfare to obey her behests with alacrity and give them your best efforts. This last advice is from personal experience.—*N. C. Medical Journal.*

